# Digital trust in gaming communities: chained mediation by perceived media realism and group identity among university students

**DOI:** 10.3389/fpsyg.2025.1691196

**Published:** 2026-01-12

**Authors:** Cui Huang, Yifu Huang, Shouchao Guo, Wenbo Deng

**Affiliations:** 1 Guangling College, Yangzhou University, Yangzhou, China; 2 School of Journalism and Communication, Yangzhou University, Yangzhou, China; 3 School of communication, Qufu Normal University, Qufu, China

**Keywords:** digital trust, gaming communities, group identity, perceived media realism, university students

## Abstract

**Introduction:**

With the rapid development of Web 3.0 and the metaverse, game communities have evolved into complex social ecosystems. In these environments, digital trust is essential for maintaining user engagement and fostering cooperation. This study aims to explore the influence of game community participation on users’ digital trust, as well as the mediating roles of perceived media realism and group identity.

**Methods:**

A cross-sectional survey was conducted among 494 university students in Jiangsu, China. Validated scales were used to measure the constructs of interest. Structural equation modeling (SEM) was employed to analyze the data and test the hypothesized relationships.

**Results:**

The results indicate that game community participation has a positive impact on digital trust. Both perceived media realism and group identity significantly mediate this relationship. Additionally, a significant chain mediation effect was observed, where perceived media realism influences digital trust through group identity.

**Discussion:**

This study provides a theoretical basis for understanding the dynamics of digital trust within game communities. The findings suggest that enhancing perceived media realism and fostering group identity can promote higher levels of digital trust among users. These insights are valuable for guiding the healthy development of gaming communities in the context of Web 3.0 and the metaverse.

## 1 Introduction

As a highly social virtual space, game communities shape complex social networks through immersive collaboration mechanisms, such as team dungeons and resource exchanges (Cole and Griffiths, 2007). Industry data shows that 67% of global players form new social connections through games, 77% believe games facilitate cross-group connections, and 60% experience a strong sense of community belonging (The Entertainment Software Association (ESA), 2023). However, issues like anonymity and virtual identity fraud pose unique challenges to building trust. Players must assess cooperation risks with limited information, and traditional institutional safeguards are less effective in such environments (Gefen et al., 2003; Spadaro et al., 2020). Therefore, understanding the mechanisms of digital trust formation in game communities is crucial for optimizing platform governance and user experience.

In the context of the rapid advancement of Web 3.0 and metaverse technologies, digital trust—the belief that others on digital platforms are reliable and trustworthy—has become a core element in maintaining the healthy ecosystem of virtual communities (World Economic Forum, 2023a). Unlike traditional trust models, digital trust is built through dynamic social exchanges and shared experiences (McKnight et al., 2002), playing an indispensable role in fostering user cooperation and enhancing participation satisfaction (Gandolfi, 2022). Particularly in online environments where anonymous interactions and virtual identities are prevalent, digital trust significantly reduces users’ risk perception, laying the foundation for sustainable community collaboration (Wu et al., 2023).

First, in terms of research context, existing literature on digital trust predominantly focuses on e-commerce, online transactions, or traditional social media platforms (e.g., Gefen et al., 2003; McKnight et al., 2002). Trust in these contexts is typically associated with institutional safeguards, transaction security, or personal information disclosure. However, game communities, as highly immersive virtual social ecosystems centered on emotional connections and collaborative tasks, exhibit unique trust mechanisms. Here, trust extends beyond the reliability of information or transactions to encompass beliefs about others’ capabilities and intentions in anonymous environments, as well as commitments to collective goals. Directly applying traditional trust models to game communities may fail to fully capture their dynamic, emotion-driven processes of trust formation. Secondly, in the exploration of mechanisms, even within game-related research, most discussions on trust remain confined to superficial analyses of influencing factors (such as interaction frequency and social norms) or treat it as a static outcome variable. The research has yet to fully reveal the psychological black box underlying the transition from ‘participation’ to ‘trust’ among users (Gandolfi, 2022).

To investigate these questions, this study constructed a theoretical model with perceived media realism and group identity as chain mediators, grounded in Media Richness Theory and Social Identity Theory. The model was tested using a cross-sectional survey of 494 university students in Jiangsu Province, China. Data were collected using validated scales and analyzed via structural equation modeling. The paper is structured as follows: the Introduction outlines the research background and questions; the Literature Review synthesizes the core constructs and theoretical foundations; the Methods section details the data collection and measures; the Results present the data analysis and findings; the Discussion interprets the results and underlying mechanisms; and the final section concludes with the implications, limitations, and future research directions.

## 2 Literature review

### 2.1 Digital trust in online communities

Digital trust represents a relational construct rooted in users’ confidence in the reliability, integrity, and predictability of other actors (individuals, systems, or institutions) within digital ecosystems (McKnight et al., 2002; World Economic Forum, 2023b). Unlike institution-based trust in physical settings, it emerges dynamically through repeated digital interactions, shared experiences, and perceived reciprocity (Gefen et al., 2003; Lu et al., 2016).

In the context of gaming communities, digital trust also exhibits a “transferable” nature. Kim and Kim (2023) proposed based on the trust transfer theory that trust in online gaming communities (including cognitive trust in reliability and affective trust in emotional attachment) can be transferred to brands advertised by the community. This is because members perceive an associative relationship between the trusted community and the unknown brand, and such trust transfer further enriches the connotation of digital trust in gaming contexts—digital trust is not only interpersonal (trust in other members) but also extends to “community-brand” relational trust (Kim and Kim, 2023).

Additionally, emphasized the connection between digital trust and psychological well-being from the perspective of self-determination theory. In their study on 704 online gamers, digital trust was found to positively correlate with “vitality” (a construct integrating physical and mental health), as trust reduces perceived risk in virtual interactions, thereby satisfying users’ psychological needs for autonomy and relatedness—this further explains why digital trust is critical for sustaining long-term community engagement.

Game communities present a unique and intensified environment for studying digital trust due to the following four inherent characteristics. First, trust in Other Players’ Behavior (Cole and Griffiths, 2007; Williams et al., 2006). Second, trust in Transaction Systems (Gefen et al., 2003; Xu et al., 2024). Third, Heightened Emotional Investment and Social Identity (Tajfel and Turner, 1979a; Postmes et al., 2000). Fourth, the Role of Media Richness and Perceived Realism (Daft and Lengel, 1986; Shen, 2015; Gonçalves et al., 2022). Overall, Digital trust in game communities is a multifaceted construct, critically dependent on competence, integrity, benevolence, and systemic reliability (Di Napoli et al., 2019). The unique combination of anonymity, high-stakes collaboration, complex virtual economies, strong identity formation, and immersive media richness creates a distinctive ecosystem where trust is both intensely demanded and uniquely shaped. Understanding these contextual specificities is crucial for designing trustworthy gaming platforms and fostering healthy virtual communities.

### 2.2 Game community participation

Game Community Participation refers to players’ behavioral and emotional investment in online games and their derivative communities. The forms of participatory behavior are diverse, including joining guilds or factions, discussing game topics in community forums or chat groups, co-creating game-related content, such as guides, mods, videos, and teaming up to complete activities like dungeons or battles. These behaviors reflect both instrumental interaction among players like sharing information, exchanging strategies, and deep collaboration like team raids, resource sharing, accompanied by players’ sense of belonging and identification with the community. For instance, Hsu and Lu (2004) noted that one primary motivation for players to engage in communities is to acquire game information, and frequent participation can enhance skill mastery and the overall gaming experience. Concurrently, Gandolfi (2022) found that the emotional dimension of game community participation is equally important, as player engagement often fosters emotional commitment to shared goals, a sense of belonging, and identity formation (Jung, 2020). Overall, game community participation is not merely a quantification of playtime or login frequency; it represents the profound attachment and emotional investment generated by players through interactions within virtual spaces.

University students are the core user group of the game community (State Council Information Office of China, 2021). This group has both technical sensitivity and social activity, and its cognitive development characteristics have a profound impact on its interpretation of media authenticity and trust decisions (Kowert and Oldmeadow, 2013). Research suggests that high-frequency collaboration in the game will strengthen the complementary role cognition and group loyalty, especially in the youth group (Teng et al., 2012; Zhong, 2011). This makes them an ideal sample for exploring the transformation chain of “cognition-society-trust.” Paying attention to this group not only helps to understand the trust logic of digital aborigines, but also provides an empirical basis for policy makers to design a “time limited guidance” framework.

### 2.3 The mediating role of perceived media realism

Perceived media realism encompasses both objective and subjective dimensions. Objectively, it reflects the fidelity and realism of the media presentation; subjectively, it captures the audience’s perceptual and emotional experience of realism (Hall, 2003). Hall (2003) identified five key facets of media realism: plausibility, typicality, factuality, narrative consistency, and perceived quality. This multidimensional framework underlines that realism involves both surface-level detail and deeper cognitive assimilation (Pouliot and Cowen, 2022). Gonçalves et al. (2021) further distinguished realism from fidelity in immersive virtual experiences, showing that objective realism (e.g., graphical detail, accurate physics) positively influences users’ sense of presence, task satisfaction, and evaluations of virtual agents. Zhang (2021) similarly found that high realism significantly enhances cognitive engagement. Our model draws on Media Richness Theory which posits that a channel’s capacity to convey social cues determines its ability to reduce communication ambiguity and foster shared understanding (Daft and Lengel, 1986). Within a trust-formation framework, perceived media realism functions both as a boundary condition and facilitative variable. First, when users perceive a highly authentic interactive environment, their inclination to trust the platform and its members increases significantly (Gefen et al., 2003). Second, realism enhances positive attributions of others’ motives, thereby amplifying trust via social exchange and identity pathways (Nabi and Oliver, 2009; Daft and Lengel, 1986).

Perceived realism also exerts broad influence on social media behaviors, virtual immersion, cross-media effects, and even problematic usage. A strong sense of realism amplifies message persuasiveness, facilitating the transfer of online attitudes and behaviors to offline contexts (Liu et al., 2021). In gaming environments, Ribbens’s (2020) six-factor model highlights that social realism and avatar embodiment are as critical as technical simulation in shaping player experiences. For instance, players who perceive high realism in guild or team modes report stronger group affiliation and cooperative intent, positively affecting retention and social conduct (Zhang, 2015). Li and Cho (2023) showed that when content is perceived as truly authentic, individuals with high social motivation are more prone to immersive—and sometimes excessive—engagement. Modern game platforms employ rich, multimodal media—such as real-time voice chat, detailed avatar expressions, and shared virtual environments—to transmit an abundance of cues (textual, visual, auditory, and behavioral) that minimize ambiguity (Dennis and Kinney, 1998). Instant feedback during cooperative tasks further enhances predictability and relational continuity (Zheng et al., 2023). High perceived realism effectively elicits social presence, the feeling that one’s partner is “actually there” in the digital space (Biocca et al., 2003), thereby promoting recognition of trustworthy behavior and emotional resonance. These illustrate the bidirectional regulatory role of realism in online ecosystems.

### 2.4 The mediating role of group identity

Group identity refers to an individual’s psychological sense of belonging to a collective, characterized by emotional attachment, self-categorization, and value internalization (Tajfel and Turner, 1979b; Hogg, 2016). In gaming communities, this construct manifests through multilayered affiliations: Game Identity、Server Identity, Guild Identity, Role Identity, Esports Team Identity and so on.

In Social Identity Theory, individuals derive self-concept and self-esteem from membership in salient groups (Tajfel, 1981; Hogg, 2016). Within game communities, players self-categorize based on shared goals, common symbols, and collective norms, thereby forming a strong in-group consciousness (Williams et al., 2006; Li and Cho, 2023). High group identity causes members to view fellow in-group players as “trusted allies,” presuming their competence and integrity while reducing perceived risk. Emotional bonds are strengthened, giving rise to supportive behaviors—such as resource sharing or emotional encouragement—that resemble “virtual kinship” (Choi et al., 2011; Nakamura, 2020; Trude, 2022).

Group identity not only fosters in-group favoritism and collective solidarity (Tajfel, 1981) but also unifies individual motives with group objectives, serving as a mediator between community participation and trust. Empirical research in virtual teams demonstrates that even in the absence of face-to-face interaction, strong group identification correlates with higher trust in collaborators (Postmes et al., 2000). Moreover, identity-driven trust exhibits a self-reinforcing cycle: positive group experiences heighten identity salience, which in turn promotes further trust-building behaviors (Dholakia et al., 2004). Meanwhile, Kim further found that financial, social, and structural bonds within gaming communities, respectively, shape cognitive and affective trust toward the community, which then transfers to brand trust (Kim and Kim, 2023).

Despite these insights, prior studies have largely overlooked the interplay between group identity and digital trust in gaming contexts. Research on institutional trust has focused on structural safeguards (Gefen et al., 2003), Most research on interpersonal trust focuses on a static “snapshot” perspective, where trust is measured as an independent, mediating, or dependent variable at a single point in time. The role of collective identity in bridging macro-level community dynamics with micro-level trust perceptions remains underexplored. By positioning group identity as a key mediator, our study fills this gap, positing that sustained participation in game communities strengthens identity, thereby reducing perceived risk and enhancing confidence in fellow members’ reliability.

### 2.5 Chain mediation of perceived media realism and group identity

Grounded in Social Identity Theory and Media Richness Theory, our framework posits a chain mediation model for digital trust formation in game communities. It proposes that community participation initially elevates perceived media realism through immersive, cue-rich interactions (Shen, 2015; Gonçalves et al., 2021). This perceived realism subsequently fortifies group identity by enhancing the authenticity and salience of the shared social environment, which ultimately becomes the primary basis for establishing digital trust (Dholakia et al., 2004; Ribbens, 2020).

Perceived media realism may itself bolster group identity. From the perspective of identity formation, digital environments such as social media provide individuals with crucial contexts for exploring and constructing their identities (Soh et al., 2024). The study on adolescent consumer identity provides strong empirical support for this. For example, social media influencers posting content that appears “behind-the-scenes” or “unfiltered” enhances the perceived authenticity and credibility of the content. This perceived realism encourages adolescents to form stronger parasocial relationships (PSRs) with these influencers and identify more deeply with the consumer groups or subcultures that the influencers represent. For example, adolescents who follow celebrities or opinion leaders on social media often report stronger pseudo-social relationships (PSRs) when consumption of more “behind-the-scenes,” unfiltered content enhances their sense of realism (Bond, 2016). Similarly, studies of political figures’ use of Twitter have found that when messages appear more “real,” recipients experience greater empathy and more favorable evaluations of both the source and its associated group—demonstrating how perceived media realism can fortify political group identity (Lee and Shin, 2014).

On the basis of these considerations, we posit that perceived media realism and group identity each mediate the link between game community participation and digital trust. However, despite these theoretical connections, the sequential interaction between perceptual media realism and group identity in the construction of trust within gaming communities has not been sufficiently examined in existing empirical studies. This unexplored sequential mechanism represents the core research gap that this study aims to address. Moreover, we anticipate that the strength of these mediation paths will vary with the level of perceived media realism: Concretely, game community engagement first shapes perceived media realism, which then affects group identity, and ultimately impacts digital trust.

### 2.6 Research hypotheses

**Figure 1:**
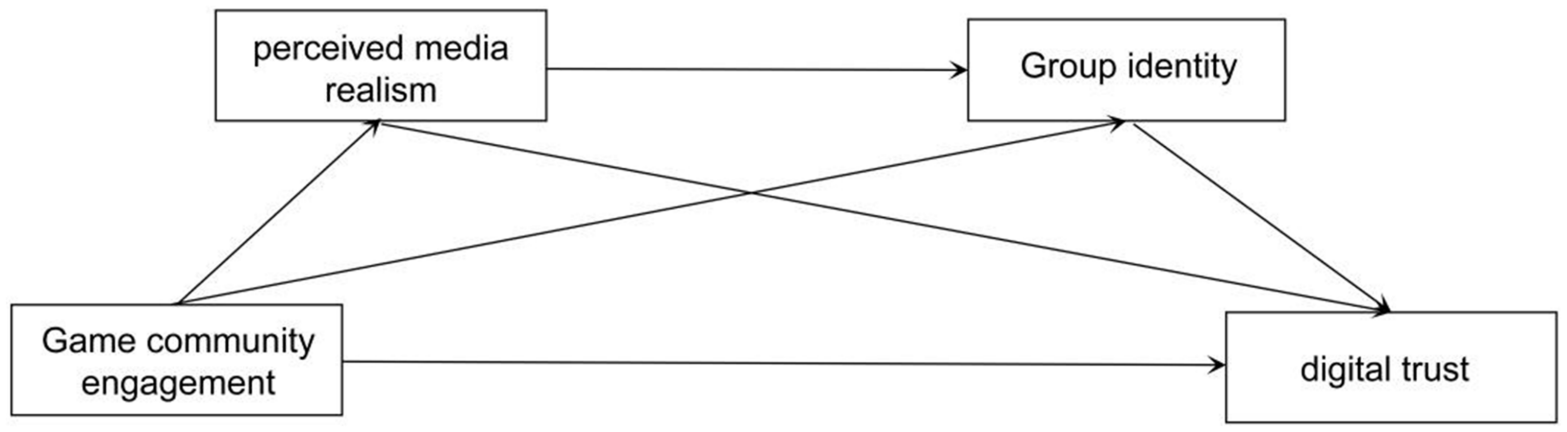
Hypothetical relationship between game community engagement, perceived media realism, group identity, and digital trust.

Based on the foregoing, we identify perceived media realism and group identity as key antecedent variables affecting digital trust in online communities. Accordingly, this study develops a chain mediation model—grounded in Social Identity Theory and Media Richness Theory—to examine how game community participation shapes digital trust via these two mediators (Figure 1). We propose hypotheses as follows:

Active participation in game communities involves repeated interactions, information sharing, and collaborative tasks. According to the literature on virtual communities, such sustained engagement is a fundamental precursor to trust, as it provides members with opportunities to observe others’ behaviors, assess their reliability, and develop expectations of reciprocity (Ridings et al., 2002; Cole and Griffiths, 2007). Therefore, we hypothesize that:

H1: Game community engagement positively affects digital trust.

Media Richness Theory (Daft and Lengel, 1986) posits that richer media, capable of conveying multiple cues and providing immediate feedback, can reduce ambiguity and foster shared understanding. In game communities, rich, multimodal interactions (e.g., voice chat, avatar expressions) enhance users’ perception of media realism. This heightened sense of realism, in turn, makes the virtual environment and social interactions feel more authentic and predictable, thereby facilitating the transfer of real-world social norms and trust heuristics to the digital context (Shen, 2015). Thus, we posit that perceived media realism serves as a cognitive mechanism translating participation into trust. We hypothesize:

H2: Perceived media realism mediates the relationship between game community engagement and digital trust.

From the perspective of Social Identity Theory (Tajfel and Turner, 1979a), active participation in a group strengthens individuals’ identification with that group. In game communities, shared goals, common symbols, and collective experiences foster a strong sense of group identity. A salient group identity leads to in-group favoritism, where members are more likely to attribute positive traits (e.g., competence, benevolence) to fellow members and perceive them as trustworthy allies (Postmes et al., 2000; Williams et al., 2006). Hence, group identity is expected to act as a social-psychological pathway from participation to trust. We hypothesize:

H3: Group identity mediates the relationship between game community engagement and digital trust.

Building on both theoretical frameworks, we propose a sequential cognitive-social process. We argue that the influence of community participation is not merely parallel through the two mediators, but sequential. First, rich interactions inherent in game communities (e.g., coordinated raids, real-time communication) enhance the perceived realism of the media environment (Gonçalves et al., 2021). This cognitively authentic experience then provides a fertile ground for social identity formation, as a realistic and immersive environment makes the shared group experience more salient and emotionally engaging (Lee and Shin, 2014). Finally, this strengthened group identity becomes the primary basis for establishing solid digital trust among members (Dholakia et al., 2004). This forms a coherent chain from media perception to social bonding and then to relational trust. We hypothesize:

H4: Perceived media realism and group identity sequentially mediate the relationship between game community engagement and digital trust.

## 3 Methods

### 3.1 Data collection and sample characteristics

A convenience sampling approach was employed to recruit 525 undergraduate students from two institutions in Yangzhou, China. At the outset of the survey, participants were informed of the study’s primary objective—to examine the relationship between game community participation and digital trust. The questionnaire was administered anonymously, and respondents were free to discontinue participation at any time. Demographic information, including gender, age, and year of study, was collected first. Participants were then asked which gaming communities they engaged with in their daily lives and for what purposes. Finally, respondents completed the instruments measuring the focal constructs. To ensure data completeness, all items were mandatory, eliminating missing responses. After pre-testing and getting the results right, we started the formal data collection. Data collection concluded in May 2025, yielding 494 valid questionnaires. The final sample comprised 249 males (50.4%) and 245 females (49.6%), with a mean age of 20.998 years (SD = 2.439).

### 3.2 Measures

#### 3.2.1 Game community participation

The Game Community Involvement Scale (Soyturk et al., 2020; Gandolfi et al., 2023) was used to assess participants’ level of engagement in game communities. This validated instrument comprises 14 items rated on a 7-point Likert scale (1 = “strongly disagree” to 7 = “strongly agree”) and captures three dimensions: community attraction, community acceptance, and community cognition. Sample items include “I am motivated to explore issues and topics related to the game community” and “Online discussions in the game community help me gain different perspectives and are valuable.” Higher mean scores indicate greater community involvement. The confirmatory factor analysis of the scale showed good fitting index (χ2 /df = 4.083, CFI = 0.914, TLI = 0.941, RMSEA = 0.079, SRMR = 0.043), and the overall consistency reliability of the scale was 0.946.

#### 3.2.2 Perceived media realism

We employed the perceived media realism scale developed by Sundar and Limperos (2013) under the Uses and Gratifications 2.0 framework, focusing on the Realism dimension. The scale consists of four items, each rated on a 7-point Likert scale (1 = “strongly disagree” to 7 = “strongly agree”). All original items underwent a translation–back-translation procedure to ensure cross-cultural validity and were contextually adapted for game community interactions (e.g., replacing “communication technology” with “game community interaction”). The confirmatory factor analysis of the scale showed good fitting index (χ^2^ /df = 4.45, CFI = 0.95, TLI = 0.95, RMSEA = 0.06, SRMR = 0.03), and the overall consistency reliability of the scale was 0.90.

#### 3.2.3 Group identity

Group identity was measured using Brown et al.’s (1986) Social Identity Scale. The scale includes five items (e.g., “Other members in the game community I participate in care about people getting along”), rated on a 7-point Likert scale. This instrument has demonstrated high internal consistency in previous research. The confirmatory factor analysis of the scale showed good fitting index (χ^2^ /df = 5.551, CFI = 0.975, TLI = 0.950, RMSEA = 0.096, SRMR = 0.029), and the overall consistency reliability of the scale was 0.702.

#### 3.2.4 Digital trust

Digital trust was measured with the 11-item Community Trust Scale proposed by Ridings et al. (2002), emphasizing community members’ perceived ability, integrity, and benevolence. In this study, “digital trust” primarily refers to trust in other members of the community, and all the scales we used were consistent with this definition. An example item is “I feel good about participating in this game community.” All items were rated on a 7-point Likert scale (1 = “strongly disagree” to 7 = “strongly agree”). The confirmatory factor analysis of the scale showed good fitting index (χ^2^ /df = 4.439, CFI = 0.961, TLI = 0.950, RMSEA = 0.083, SRMR = 0.031), and the overall consistency reliability of the scale was 0.910.

## 4 Preliminary analysis

### 4.1 Community platform usage

To assess participants’ gaming community usage, we provided a multiple-choice question listing major platforms. The most widely used game communities were composite platforms (e.g., Tencent Game Community, NGA, Steam, TapTap), selected by 69.64% of respondents. Company-specific or platform-specific communities (e.g., MiHoYo Community, NetEase Open Platform, Xiaoheihe) were used by 43.93%, and other general communities (e.g., Baidu Tieba, Bilibili) by 42.31%. In terms of frequency, 50.61% of participants accessed game strategies and tips (57.29%), obtaining game news and updates (58.50%), and finding like-minded peers or interest groups (40.08) in Table 1.

**Table 1:** Descriptive statistics and correlation coefficients of variables (N = 494).

Variable	1	2	3	4	Mean value	Standard deviation
Group identity	—				4.552	0.989
Game community engagement	0.626**	—			4.851	0.989
Digital trust	0.628**	0.456**	—		4.655	0.857
Perceived media realism	0.404**	0.350**	0.480**	—	3.982	1.060

*p < 0.05, **p < 0.01.

### 4.2 Correlation analysis

Means, standard deviations, and Pearson correlation coefficients among game community participation, perceived media realism, group identity, and digital trust are reported in Table 2. Analyses were conducted in SPSS 26.0. Digital trust correlated positively with community participation (r = 0.54, *p* < 0.01), perceived media realism (r = 0.55, *p* < 0.01), and group identity (r = 0.70, *p* < 0.01). Group identity was also positively associated with perceived media realism (r = 0.41, *p* < 0.01) and community participation (r = 0.66, *p* < 0.01). Finally, perceived media realism correlated positively with community participation (r = 0.53, *p* < 0.01). These results support hypotheses H1–H4.

**Table 2:** Summary of the effect analysis process.

Effect	Term	Effect	*SE*	*t*	*p*	LLCI	ULCI
Direct effect	Game community engagement ⇒ digital trust	0.122	0.047	2.610	0.009	0.030	0.213
Indirect effect process	Game community engagement ⇒ Perceived media realism	0.796	0.053	14.898	0.000	0.691	0.900
	Game community engagement ⇒ Group identity	0.601	0.042	14.452	0.000	0.519	0.682
	Perceived media realism ⇒ Group identity	0.310	0.032	9.770	0.000	0.248	0.372
	Perceived media realism ⇒ digital trust	0.231	0.032	7.201	0.000	0.168	0.293
	Group identity ⇒ digital trust	0.376	0.046	8.104	0.000	0.285	0.466
gross effect	Game community engagement ⇒ digital trust	0.623	0.037	16.786	0.000	0.551	0.696

LLCI refers to the lower limit of the 95% confidence interval, and ULCI refers to the upper limit of the 95% confidence interval.

### 4.3 Chain mediation analysis

**Figure 2:**
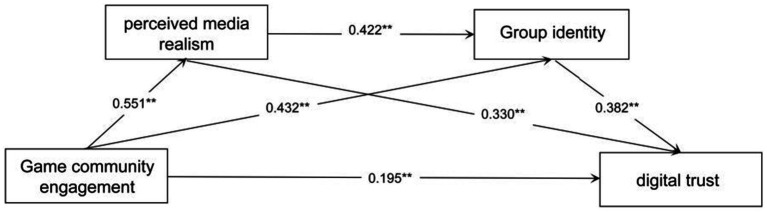
The proposed sequential mediation model. Path values are the path coefficients. All path coefficients were standardized. *p < 0.05, ***p < 0.001.

We tested the proposed chain mediation model using Hayes’s PROCESS macro (Model 6) in SPSS 26.0, with 5, 000 bootstrap resamples to generate bias-corrected 95% confidence intervals (CIs) for indirect effects (Hayes, 2018). Demographic variables were controlled in all models, and four mediation paths were examined. The total effect of community participation on digital trust was significant (total effect = 0.623, 95% BCa(Bias-Corrected and Accelerated) CI [0.551, 0.696]). The direct effect remained significant when mediators were included (direct effect = 0.122, 95% BCa CI [0.030, 0.213]). Three specific indirect paths were then tested: Perceived media realism as Mediator: Community participation → perceived media realism → digital trust yielded a significant indirect effect of 0.183 (95% BCa CI [0.133, 0.263]), supporting H3a. Group Identity as Mediator: Community participation → group identity → digital trust produced an indirect effect of 0.226 (95% BCa CI [0.158, 0.321]). Chain Mediation: Community participation → perceived media realism → group identity → digital trust demonstrated a significant chain indirect effect of 0.093 (95% BCa CI [0.062, 0.137]). The total indirect effect (sum of all three mediating paths) was also significant (total mediated effect = 0.502, 95% BCa CI [0.439, 0.609]). All three hypotheses were supported. Results are detailed in Table 3 and illustrated in Figure 2. In summary, we found that perceived media realism and group identity each function as independent mediators of the effect of game community participation on digital trust. Crucially, they also operate in sequence: participation enhances perceived media realism, which in turn fosters group identity, ultimately increasing digital trust.

**Table 3:** Analysis of indirect effects.

Term	Effect	Boot SE	Boot LLCI	Boot ULCI	*z*	*p*
Game community engagement ⇒ Perceived media realism ⇒ digital trust	0.183	0.033	0.133	0.262	5.611	0.000
Game community engagement ⇒ Group identity ⇒ digital trust	0.226	0.042	0.158	0.321	5.422	0.000
Game community engagement ⇒ Perceived media realism ⇒ Group identity ⇒ digital trust	0.093	0.019	0.062	0.137	4.799	0.000

BootLLCI refers to the lower limit of 95% interval of bootstrap sampling, and Boot ULCI refers to the upper limit of 95% interval of bootstrap sampling. Bootstrap type: percentile bootstrap method.

## 5 Discussion

Given the increasing complexity of interpersonal dynamics in digital environments, elucidating the mechanisms underpinning digital trust is paramount. This study investigated how participation in game communities influences users’ digital trust levels, specifically examining the mediating roles of perceived media realism and group identity. Drawing on media richness theory and social identity theory, we constructed and tested a chain mediation model using survey data from 494 university students.

### 5.1 The gaming community directly impacts digital trust

The findings of this study explicitly support Hypothesis H1, confirming that game community participation is associated with a small but significant direct relationship with digital trust (direct effect = 0.122, 95% BCa CI [0.030, 0.213], *p* < 0.05; total effect = 0.623, 95% BCa CI [0.551, 0.696], *p* < 0.01). Correlation analysis further reinforces this relationship, with a strong positive correlation between participation and digital trust (r = 0.54, *p* < 0.01). This result underscores the foundational role of active community engagement in fostering digital trust.

The positive correlation between game community participation and digital trust (r = 0.54) and the significant total effect (β = 0.623) in our model align with prior literature, which identifies sustained engagement as a key antecedent of trust in virtual communities (Ridings et al., 2002). This relationship is understood to develop through repeated interactions, the establishment of shared norms, and the gradual accumulation of perceived reliability (Cole and Griffiths, 2007; Wu et al., 2023). Our findings extend this understanding by revealing that in the context of game communities, this influence operates primarily through indirect psychological pathways—namely, by enhancing the perceived realism of the environment and fostering a strong group identity. The minimal direct effect observed after accounting for these mediators suggests that the primary value of participation lies in its capacity to create a rich, socially salient environment wherein the cognitive and social foundations for trust can be established.

This participation both shapes social roles and deepens interpersonal interactions within the community. Players often collaborate through division of labor in guilds, teams, or factions, forming distinct social roles and statuses. Research indicates that in role-playing online games, high-frequency teamwork can prompt players to develop complementary role perceptions and strong guild loyalty. For instance, in MMORPGs such as World of Warcraft, players who engage in high-frequency collaboration—averaging 53 interactions per day—develop complementary role cognition and strong guild loyalty (Cole and Griffiths, 2007; Zhang, 2011). Furthermore, game communities provide players with communication platforms that extend beyond the game itself. In large online communities, players discuss not only game strategies and techniques but also share personal life experiences, hobbies, and interests. This interaction, based on shared virtual experiences, helps forge deep emotional bonds. On one hand, this enriches the gaming experience; on the other, it promotes the enhancement of players’ real-world social skills (Saldanha et al., 2023). As players spend time together in-game, they gradually come to view each other as “comrades-in-arms” or “virtual kin,” developing strong trust and a sense of belonging towards their peers, manifested in supportive behaviors like mutual encouragement and resource sharing. This process deepens group identity awareness, leading players to develop a high level of identification with their guild or team, thereby increasing their psychological investment.

### 5.2 The mediating role of perceived media realism

The results of this study strongly support H2, confirming that perceived media realism plays a significant positive mediating role in the relationship between game community participation and digital trust. Specifically, the indirect effect of the path is 0.183 (95% BCa CI [0.133, 0.262], *p* < 0.001), indicating that perceived media realism serves as a critical bridge through which participation behavior translates into enhanced digital trust. This finding aligns with the core assertions of media richness theory (Daft and Lengel, 1986) and further clarifies the unique mechanism of trust formation in the immersive context of game communities.

The mediating mechanism of perceived media realism can be elaborated through two interconnected processes. First, active participation in game communities amplifies users’ perception of media realism. Game community participation involves diverse interactive behaviors—such as real-time voice communication during team battles, collaborative exploration of virtual landscapes, and emotional expression through avatar gestures—which expose users to multimodal information cues (verbal, visual, and behavioral). These rich cues reduce ambiguity in communication (e.g., tone of voice conveys sincerity, avatar movements reflect cooperation intentions) and enhance the sense of “social presence” (Biocca et al., 2003), making users feel that the virtual environment and interactions are “authentic” and “lifelike.” For example, frequent participation in guild activities with high-quality voice chat and detailed character animations may lead players to perceive their teammates as “real and present,” thereby strengthening their sense of media realism. Second, heightened perceived media realism is significantly linked to digital trust. A potential mechanism, drawn from theory (Shen, 2015), is that when users perceive the environment as realistic, they may be more likely to transfer real-world social norms and trust criteria to the virtual context. *It is speculated* that a higher sense of realism may reduce the salience of the ‘virtual vs. real’ psychological barrier. However, this specific mediating pathway of barrier reduction was not directly measured in our study and warrants verification in future research, for instance, by experimentally manipulating media realism making users attribute others’ behaviors to stable personality traits rather than temporary or deceptive acts. Theoretically, this cognitive shift could lower perceived interaction risks and facilitate more positive evaluations of others’ competence, integrity, and benevolence—three core dimensions of digital trust (Ridings et al., 2002; Gefen et al., 2003).

This mediating effect highlights the uniqueness of game communities compared to text-based or low-immersion virtual platforms. As noted in the study, modern game communities leverage multimodal media (voice, avatar expressions, shared virtual spaces) to create a highly realistic interaction environment. This richness is associated with faster trust formation compared to contexts with limited cues. It is plausible that perceived realism contributes to this by simulating the immediacy of face-to-face communication (Gonçalves et al., 2022), thereby potentially shortening the “trust-building period.” Thus, the mediating role of perceived media realism explains why users who actively participate in interactive, high-fidelity game communities tend to develop stronger digital trust: their participation enhances the realism of the environment, which in turn fosters trust through more authentic and predictable social interactions.

### 5.3 The mediating role of group identity

The findings of this study provide robust support for Hypothesis H3, confirming that group identity significantly mediates the relationship between game community participation and digital trust. Specifically, the indirect effect of the pathway is 0.226 (95% BCa CI [0.158, 0.321], *p* < 0.001), indicating that group identity serves as a critical psychological bridge through which participation in game communities translates into enhanced digital trust. This result aligns with the core tenets of social identity theory (Tajfel and Turner, 1979b) and sheds light on the social-psychological mechanisms underlying trust formation in virtual gaming contexts.

Game community participation plays a pivotal role in shaping group identity and emotional investment. As participation increases, so do players’ feelings of identification and belonging towards the community, prompting individual behavior to align with group goals. According to Social Identity Theory (Tajfel and Turner, 1979a), individuals derive self-concept and self-esteem from group affiliations. In game communities, shared symbols and norms—such as guild emblems, exclusive terminology, and team rules—strengthen players’ sense of belonging, leading them to perceive peers as “trustworthy ingroup members.” This strong group identity is correlated with heightened in-group favoritism; players reporting stronger identity were more inclined to believe that fellow group members share the same values and cooperative intentions, a perception which is, in turn, associated with lower levels of mutual suspicion and distrust. For instance, research shows that even in virtual team environments lacking face-to-face contact, higher levels of group identity correlate with higher levels of trust among members (Postmes et al., 2000). Simultaneously, a strong sense of identity motivates players to engage in positive behaviors like helping teammates and sharing resources, further reinforcing group cohesion and emotional investment. This “virtual kinship” relationship, built on shared goals and experiences, endows game communities with characteristics akin to intimate social groups in the real world, providing players with social support and psychological fulfillment often difficult to attain in reality.

This mediating effect underscores the unique dynamics of game communities compared to other virtual spaces. Unlike generic social media platforms, game communities often revolve around collaborative, goal-driven activities (e.g., team quests, guild wars) that inherently strengthen group identity. These activities create “shared fate” scenarios, where individual success depends on group performance, intensifying the link between identity and trust (Williams et al., 2006). For instance, in a raid requiring precise role coordination, repeated participation not only builds group identity but also reinforces the belief that “we rely on each other,” thereby elevating trust in teammates’ competence and commitment.

The findings also extend prior research on virtual trust. While studies like Dholakia et al. (2004) noted that group identity correlates with trust in online communities, the current study specifies this relationship in gaming contexts, highlighting that group identity mediates the effect of participation on trust. This is particularly relevant for university students, the sample in this study: as a socially active demographic, they are more likely to form strong group bonds through game participation, and these bonds directly shape their trust judgments in virtual interactions (Kowert and Oldmeadow, 2013).

### 5.4 Analysis of chain mediation effect between perceived media realism and group identity

The results of this study strongly support Hypothesis H4, confirming that perceived media realism and group identity play a significant chain mediating role in the relationship between game community participation and digital trust. Specifically, the chain indirect effect of the pathway “game community participation → perceived media realism → group identity → digital trust” is 0.093 (95% BCa CI [0.062, 0.137], *p* < 0.001), indicating that the two variables jointly form a sequential mediating mechanism, through which game community participation indirectly influences digital trust.

Prior research suggests that a strong sense of presence or immersion in media environments can deepen users’ emotional connection to a group, thereby increasing identification and cooperative tendencies (Lee and Shin, 2014). Our findings extend this understanding by demonstrating that the perceived realism of the communication environment acts as a critical cognitive precursor. It precedes and facilitates the social psychological process of group identity formation, which then serves as the primary driver of trust. This delineates a clear cognitive process. Consequently, our model suggests that a technologically rich communication environment (which enhances perceived realism) and a strong sense of group cohesion are associated with the cultivation of robust digital trust.

The most significant theoretical insight from our model is the identification of a robust chain mediation pathway: Game community participation enhances perceived media realism, which subsequently fosters stronger group identity, ultimately leading to increased digital trust. This sequential effect is theoretically grounded. In theory, perceived reality can activate users’ cognitive mechanisms by making the environment appear “real,” thereby triggering the formation of identity in deeper social psychological processes; Only after this will this identity be transformed into relational trust. This staged development process (cognition → society → relationships) provides a more detailed understanding than parallel or single mediation models, highlighting that platforms must first create authentic and immersive interactive experiences in order to stimulate the identity recognition process that ultimately generates trust.

Taken together, these findings offer a significantly more comprehensive framework for understanding digital trust formation in immersive online communities. While existing research often highlights the role of repeated behavioral engagement (Fu, 2020), our study reveals deeper, interlinked cognitive and social mechanisms. We provide robust empirical evidence for a chain mediation model where participation’s impact flows sequentially through perceived media realism and then group identity before culminating in trust. This model addresses a key limitation in prior work by integrating the media theory (Daft and Lengel, 1986) and social identity theory (Tajfel and Turner, 1979b), demonstrating their synergistic roles as mediators. Ultimately, we advance a holistic view of trust formation in virtual environments, emphasizing the dynamic interplay between user engagement, cognitive perception of the media environment, and the development of social group bonds. Furthermore, while this study primarily reveals the positive role of perceived media realism in building digital trust, the relationship between realism and user experience may not be a simple linear positive correlation. Future research could further explore whether, in specific contexts such as interactions with highly humanlike yet imperfect avatars, excessive realism might trigger negative reactions like the ‘uncanny valley effect,’ thereby creating new boundary conditions for trust establishment.

## 6 Conclusion

In an era where digital media pervade everyday life, game communities have transcended their role as mere entertainment venues to become crucial platforms for digital natives—especially adolescents—to forge social ties, seek identity affirmation, and satisfy emotional belonging. Grounded in Social Identity Theory and the construct of perceived media realism, this study empirically examined how game community participation shapes digital trust via two mediators: perceived media realism and group identity. The results demonstrated that participation not only directly enhances users’ digital trust but also operates through a chain mediation pathway—first by amplifying perceived media realism, which in turn strengthens group identity, ultimately fostering trust.

### 6.1 Implications

This study offers clear practical value. First, in light of increasingly complex online social environments and concerns over adolescent mental health, establishing stable and trustworthy digital interaction mechanisms is a critical component of social governance. Our findings inform the development of engaging yet healthy online ecosystems that “educate through entertainment.” Second, the central mediating role of group identity in trust formation underscores the importance of cultivating virtual community cultures that reinforce shared values and collective responsibility among youth. Finally, perceived media realism—as a subjective experience elicited by platform design and technological implementation—significantly enhances positive attributions of others’ motives. This insight offers platform developers a novel design perspective: realism extends beyond visual realism to underpin the credibility of online relationships.

As Postmes et al. (2000) observed, “norms in digital media are not static—they are continuously constructed and reconstructed through interaction.” Game communities serve as microcosms of this “construct–reconstruct” process. We contend that a deeper understanding of perceived media realism and group identity will not only facilitate trust generation but also chart a path toward building more resilient, empathetic, and responsible digital communities.

### 6.2 Limitations and future directions

This research has several limitations. First, our sample consisted predominantly of university students from eastern China, a group characterized by high education levels and technology proficiency; the generalizability of these findings to populations with lower digital literacy or in cross-cultural contexts remains to be tested (Kowert and Oldmeadow, 2013). Then, although our model and theory support this sequence, there may be theoretical reverse or parallel relationships, which could be further verified by longitudinal or experimental designs in future studies.

Limitation of this study is its cross-sectional design. Although the proposed directions of influence are theoretically justified, the data cannot conclusively demonstrate causality or rule out alternative explanations, such as reverse causality. For example, it is plausible that pre-existing digital trust might also influence one’s level of community participation or perception of media realism. It is suggested that future research should adopt longitudinal tracking design or experimental research. And regarding the measurement models, all scales were within acceptable thresholds, but RMSEA for some models, approached or fell within the ‘moderate’ fit range (e.g., 0.079–0.096). This is especially noted for the Group Identity scale (RMSEA = 0.096). This suggests that while the scales possessed good structural validity for the purposes of this study, there might be residual covariance among items or specific contextual factors not fully captured by the original factor structures. This could be attributed to its generic nature when applied to the highly specific social context of gaming communities, where group affiliations are multi-layered and complex (Williams et al., 2006). Future research could benefit from developing and validating a group identity scale tailored specifically to the unique dynamics of game-based communities.

Finally, the proposed model is predicated on the assumption of generally healthy community environments. We acknowledge that in ‘toxic’ communities rife with malicious behavior and negative social interactions, participation frequency could correlate negatively with digital trust, rather than positively (Zhu et al., 2022). Understanding how negative community dynamics disrupt the positive psychological mechanisms identified herein represents a valuable direction for future research.

## Data Availability

The raw data supporting the conclusions of this article will be made available by the authors, without undue reservation.
