# Supplementary material for: Digital trust in gaming communities: chained mediation by perceived media realism and group identity among university students
Source: Front Psychol. 2026 Jan 12;16:1691196. doi: 10.3389/fpsyg.2025.1691196 (PMC12833012; doi:10.3389/fpsyg.2025.1691196)
Supplement: Supplementary file 1 [file Data_Sheet_1.docx]

**Appendix：**

Questions on Basic Information and Game Community Usage

1. What is your gender? (Single-choice question)

Options: Male, Female

2. What is your academic year? (Single-choice question)

Options: Freshman, Sophomore, Junior, Senior, 1st-year graduate student, 2nd-year graduate student, 3rd-year graduate student, Others (please specify: ______)

3. What is your actual age? (Please enter a number directly) (Fill-in-the-blank question)

Note: Data for this fill-in-the-blank question can be obtained by downloading detailed data.

4. Which game communities do you mainly use? (Multiple-choice question)

Options:

Comprehensive game communities (e.g., Haoyouxi.com, Tencent Game Community, NGA Player Community, Steam, TapTap)

Game communities of specific companies or platforms (e.g., Mihoyo Community, NetEase Dashen, Xiao Hei He)

Other game communities (e.g., Baidu Tieba, Bilibili)

Others (please specify: ______)

5. How frequently do you use game communities? (Single-choice question)

Options: Multiple times a day, Once a day, 3-5 times a week, 1-2 times a week, Occasionally

6. What is your main purpose for participating in game community platforms? (Multiple-choice question)

Options:

Discuss game strategies/skills

Obtain game news/update information

Share creative content

Trade game items/accounts

Provide feedback/seek technical support

Find like-minded people/interest groups

Others (please fill in: __________)

7. How long have you been participating in game communities? (Single-choice question)

Options: Less than 12 months, 1-2 years, 2-5 years, More than 5 years

8. What is your role orientation in the community? (Single-choice question)

Options:

Content creator (strategies/live streams/videos, etc.)

Active discussant

Information browser

Community manager

Others: ________________

Likert Scale Questions (1 = Strongly Disagree, 7 = Strongly Agree)

Question 9: Matrix Scale Question (Average Score: 5.1)

1. The activities and discussions in the game community stimulate my curiosity;

2. I am motivated to explore issues and topics related to the game community;

3. The questions raised and discussions increase my interest in the game community;

4. The online discussions in the game community help me develop a sense of cooperation;

5. The online discussions in the game community help me gain different perspectives, which are very valuable;

6. I feel comfortable interacting with other members of the game community;

7. I feel comfortable participating in discussions in the game community;

8. In the context of the game community, I feel comfortable communicating through online media;

9. In the game community, I can freely express different opinions and still maintain mutual trust with other members;

10. I feel that my opinions are recognized by other members of the game community;

11. I have come up with practical solutions to address challenges and problems in the game community;

12. I can describe methods to test and apply the knowledge generated from discussions and activities in the game community;

13. I can apply the knowledge generated from discussions and activities in the game community to work or other activities unrelated to the game community;

14. Brainstorming and searching for relevant information have helped me solve problems related to the game community.

Question 10: Matrix Scale Question

1. I have a good feeling about the game community I participate in;

2. I identify with other members of my game community;

3. I am similar to other members of my game community;

4. My game community is an important part of my identity;

5. I want to continue cooperating with my game community;

Question 11: Matrix Scale Question

1. Other participants in the game community I participate in care a lot about people’s ability to get along with each other;

2. Other participants in the game community I participate in will not intentionally do anything to disrupt conversations;

3. Participants in the game community I participate in care about things that are important to others;

4. Participants in the game community I participate in will do their best to help others;

5. Participants in the game community I participate in strive to treat each other fairly;

6. Other participants in the game community I participate in behave inconsistently;

7. I have great confidence in the skill level of other players in my game community regarding the topics we discuss;

8. Other players in my game community have rich knowledge about the topics we discuss;

9. Other players in my game community have professional capabilities that can add value to discussions;

10. Other players in my game community are very professional in the topics we discuss;

11. Other players in my game community perform very well in tasks related to the topics we discuss.

Question 12: Matrix Scale Question

1. When interacting in the game community, I am certain that the content is real and not fabricated;

2. The interaction method of the game community is similar to face-to-face communication;

3. The user experience of the game community is highly similar to real life;

4. The game community enables me to have an immersive and realistic experience.
